# THE EVOLUTION OF FILIAL PIETY TO INTERGENERATIONAL SUPPORT IN TAIWAN

**DOI:** 10.1093/geroni/igae098.3742

**Published:** 2024-12-31

**Authors:** Yunann Lin, Hui-Ching Weng, Wang Chih-Liang

**Affiliations:** National Chung Cheng University, TAINAN, Tainan, Taiwan (Republic of China); National Cheng Kung University, Tainan City, Tainan, Taiwan (Republic of China); Spring Sun, Spring Sun clinic, Tainan, Taiwan (Republic of China)

## Abstract

**Objective:**

The practice of filial piety concept may vary with sociocultural transformation under aging impact. It’s crucial to interpret how filial behaviors affect parent–child interaction. Thereby, this study aimed to explore associations between filial piety and intergenerational support in Taiwan.

**Methods:**

This longitudinal study analyzed data collected from the 2016 Taiwan Social Change Survey, consisting of demographics, self-rated health, sibling number, children number, coresident with parents or children, reciprocal filial piety (RFP), authoritarian filial piety (AFP), financial, household, and emotional support. Participants were categorized into three cohorts: the traditional generation (born before 1945), the baby boomer generation (1946-1964), and Generation X (1965-1980). ANOVA and multilevel regression were adopted to analysis intergenerational associations. Findings: AFP present significant decrease in the baby boomer (2.55 vs 2.9, p< 0.001) and generation X (2.4 vs 2.9, p< 0.001) when comparing with the traditional generation; while RFP show no difference among three cohorts. After adjusting gender, education, marriage, self-rated health, income, sibling number, and coresident with parents, RFP of generation X was positively associated with household (p=0.05) and emotional support (p< 0.001) to parents. Moreover, AFP of baby boomer generation was marginal associated with financial (p=0.053) and household (p=0.058) support to parents.

**Conclusion:**

The diminish of AFP embodied the trend of mutual affection and equality in the parent–child relationship. The mediation effect of RFP in generation X was practiced in household and emotional support; while the mediation effect of AFP in baby boomer generation was practiced in financial and household support.

